# Hermione Exchange Educational Program: How to Integrate Multidisciplinary Approaches to Manage HR+/HER2- Metastatic Breast Cancer

**DOI:** 10.3390/cancers18132087

**Published:** 2026-06-27

**Authors:** Marina Elena Cazzaniga, Nicola Fusco, Alessandra Fabi, Umberto Malapelle, Paolo Vigneri

**Affiliations:** 1 Phase 1 Research Unit, Fondazione IRCCS San Gerardo dei Tintori, 20900 Monza, Italy; 2 Dipartimento di Medicina e Chirurgia, Università degli Studi Milano Bicocca, 20126 Milano, Italy; 3 Dipartimento di Medicina e Chirurgia, Università degli Studi Milano Statale, 20122 Milano, Italy; nicola.fusco@ieo.it; 4 European Oncology Institute, 20141 Milano, Italy; 5 Policlinico Gemelli, 00168 Roma, Italy; alessandra.fabi@policlinicogemelli.it; 6 Dipartimento di Sanità Pubblica, Università degli Studi Federico II, 80138 Napoli, Italy; umbertomalapelle@gmail.com; 7 Oncology Unit, Humanitas Catania, 95045 Catania, Italy; paolo.vigneri@humanitascatania.it; 8 Oncology Unit, Humanitas University, 20089 Rozzano, Italy

**Keywords:** endocrine sensitivity, elacestrant, luminal metastatic breast cancer, liquid biopsy targeted therapy, chemotherapy

## Abstract

Background/objective: Given the increasing complexity of the luminal breast cancer landscape, a proper characterization is required in everyday clinical practice, and the recurrence after the standard first-line treatment with CDK4/6 inhibitors with/without endocrine therapy should be managed. Method: The Hermione Exchange Educational Program was held in Milan, Italy, between September 2024 and January 2025. Two questionnaires were proposed regarding the use of targeted treatment or chemotherapy after progression from CDK4/6 inhibitors. The lecture and use cases enhanced the discussion during the workshops. Results: From the surveys, it emerged that most participants (69%) considered liver metastases at CDK4/6-inhibitor progression as a key reason to initiate chemotherapy, while lung progression influenced this choice for 50% of participants. Liver involvement guided the use of targeted therapy for 56%, and attitudes were divided on whether the duration of first-line CDK4/6 therapy should affect decisions (44% in agreement vs. 38% in disagreement). The willingness of patients to receive chemotherapy (88%) and comorbidities (81%) were significant drivers. Almost all participants agreed that both the duration of response and the molecular status were key aspects to consider when choosing a second line of therapy, along with the general clinical condition of the patient. In the lecture, tissue and liquid biopsy are considered powerful tools to describe tumor molecular features over time; such complexity should be harnessed by a close dialogue between oncologists, molecular biologists, and pathologists to optimize the therapeutic choice according to the mutational status of patients. The use cases illustrate three patients with visceral progression, non-visceral progression within 12 months, and non-visceral progression after 12 months following CDK4/6 inhibitors. Conclusion: Genomic testing should be considered at diagnosis and repeated during treatment to monitor the disease. The clinical experience acquired over the years must be integrated with new molecular knowledge.

## Simple Summary

The treatment of hormone-receptor–positive (HR+) metastatic breast cancer has become more complex in recent years. Because of this, doctors need better ways to understand each patient’s tumor and to decide what to do when the cancer stops responding to standard first-line treatments such as CDK4/6 inhibitors. To explore these challenges, a group of oncologists and molecular pathologists met in Milan between September 2024 and January 2025 as part of the Hermione Exchange Educational Programme. They discussed how breast cancer changes over time at the molecular level and how these changes might guide treatment decisions. They also reviewed real patient cases and completed questionnaires about treatment choices after CDK4/6 inhibitor failure. Their answers showed that doctors’ opinions shifted once a new drug, elacestrant, became available. The experts noted that both tissue biopsies and liquid biopsies (blood tests that detect tumor DNA) can be valuable tools. These tests help track how the cancer evolves and reveal new mutations that may influence which treatment is most likely to work. Good communication between oncologists, molecular biologists, and pathologists is important to interpret these results and choose the most appropriate therapy. They also highlighted how well a patient responded to CDK4/6 inhibitors—and for how long—can help inform what treatment to consider next. In conclusion, the group suggested that genomic testing at diagnosis and at key points during treatment may help monitor how the disease changes over time. They emphasized that clinical experience remains important, but it may need to be combined with emerging molecular information to support treatment decisions.

## 1. Introduction

Breast cancer is the most frequent cancer type among women: the estimated incidence in Italy in 2024 was just under 54,000 cases, with an estimated five-year survival rate of more than 88%, with 925,000 women living with a breast cancer diagnosis [1].

All breast cancer therapy guidelines [2,3,4] include the quantification of steroid hormone receptors (HR) for estrogen and progesterone at diagnosis in the routine clinical practice. The estrogen receptor (ER) is one of the most important biomarkers in breast cancer; it is expressed in roughly 80% of cases and is regarded as a strong prognostic factor and a key indication of responsiveness to endocrine therapy (ET). The progesterone receptor (PgR) has two isoforms (alpha and beta), is a transcriptional target of the ER, and is substantially estrogen dependent. However, it also affects ERα function in breast cancer. PgR-positive breast cancers are less common than ER-positive breast cancers, accounting for only around 20% of cases [5,6].

In the metastatic setting, endocrine therapies combined with cyclin-dependent kinase (CDK) 4/6 inhibitors continue to represent the therapeutic backbone [7]. Endocrine agents are often administered together with additional targeted treatments, such as inhibitors of phosphatidylinositol-3-kinase (PI3K), Akt serine/threonine kinase (AKT), and/or the mechanistic target of rapamycin (mTOR) pathway [7,8,9,10].

Several new approaches are also emerging and may soon be incorporated into routine treatment algorithms [11]. Camizestrant, a next-generation selective estrogen receptor degrader (SERD) and full ER antagonist [12], has demonstrated superior progression-free survival compared with aromatase inhibition in patients with ER-positive, HER2-negative advanced breast cancer harboring ESR1 mutations that developed during therapy, while maintaining CDK4/6 inhibition in the first-line setting [13].

Upon the development of initial resistance, palliative chemotherapy is typically recommended [14]. Evidence is also growing for antibody–drug conjugates (ADCs), such as trastuzumab deruxtecan (T-DXd), which have shown improved progression-free survival compared with chemotherapy in patients with hormone receptor-positive, HER2-low or HER2-ultralow metastatic breast cancer previously treated with at least one line of endocrine-based therapy. These agents have the potential to significantly reshape the therapeutic landscape of metastatic breast cancer, particularly for tumors classified as HER2-low or HER2-ultralow [15].

Resistance to ET is a significant clinical challenge. Under the selective pressure of ET, acquired activating mutations in Estrogen Receptor 1 (ESR1) lead to constitutive activation and diminish the effectiveness of aromatase inhibitors; epigenetic changes, the presence of FOXA1, aberrant c-Myc expression, and cyclin D1 activity are other mechanisms that determine ET resistance, each in a specific way [16,17]. The aberrant activation of the PI3K/AKT/mTOR pathway, driven by PI3K mutations, can lead to increased ER transcriptional activity and enhanced cell survival, thereby reducing the effectiveness of endocrine therapies. Mutations occur in up to 40% of breast cancers and predict response to PI3K inhibitors [18].

Genomic alterations play a key role in guiding treatment sequencing within clinical recommendations, particularly from the second line onward. The ESMO Precision Medicine Working Group has recently updated the classification of several biomarkers: both ESR1 and PIK3CA mutations are considered ESCAT IA. Current guidelines advise performing molecular testing after progression on first-line endocrine therapy in patients with HR-positive/HER2-negative advanced breast cancer, in order to select the most appropriate endocrine options and determine whether targeted agents should be incorporated into subsequent treatment lines [2,8].

Given the increasing complexity of the luminal breast cancer landscape, it is important to properly characterize it in everyday clinical practice and manage the recurrence after the standard first-line treatment with CDK4/6 inhibitors with/without ET.

Here, we present the findings that emerged from three workshops in the context of the Hermione Exchange Educational Program, held in Milan in April and September 2024 and January 2025.

## 2. Materials and Methods

The HERMIONE Educational Exchange Program was designed to promote scientific knowledge and the exchange of clinical experiences in the management of HR+ metastatic breast cancer. The project was developed through a structured and modular methodology, aiming to foster interaction between experts and young oncologists.

We first organized a training course, which was divided into three thematic modules:

1. Understanding the Complexity of HR+ Breast Cancer: This module provided participants with an in-depth analysis of the preclinical and clinical aspects of the disease.
2. Critical Analysis of available Therapeutic Options: Participants critically examined data from the most recent clinical studies to identify the most appropriate therapeutic strategies based on patient profiles and treatment lines.
3. Sharing Clinical Experience: This module allowed participants to share experiences gained in clinical practice and trials, promoting the comparison of therapeutic approaches. Each participant had the opportunity to present and discuss 2–3 clinical cases, benefiting from the exchange with the Scientific Committee.

### 2.1. Participants

In January 2024, the chair (CME) contacted two molecular pathologists (FN and MU) and two medical oncologists (FA and VP) to constitute the Scientific Committee. All members had direct clinical expertise on breast cancer management and were key opinion leaders in their field with at least 5 indexed publications in the last 5 years on HR+ metastatic breast cancer. Furthermore, they worked in academic hospitals or Cancer Centers located in different Italian regions and could ensure a comprehensive insight into the national situation. The Scientific Committee subsequently identified 60 clinical experts in breast cancer management to provide expert lectures on different topics and share use cases for critical discussion. The aforementioned clinicians served as speakers at some meetings and as participants at others. All but six participated in the voting process: specifically, 19 clinicians (including experts and participants) voted at the first meeting, whereas 26 clinicians voted at the second meeting.

### 2.2. Methodology

A kick-off meeting of the Scientific Committee was organized to define contents and methods; then, all participants attended an introductory virtual meeting that presented objectives, milestones, and methodology. In the subsequent 2 months, participants selected virtual clinical cases to be discussed in the dedicated modules. During the three workshops, the discussion was promoted through three methodologies: a plenary session on the diagnostic role of molecular characterization of HR+ metastatic breast cancer, the administration of a structured survey, and use cases.

### 2.3. Structured Survey

After revising the literature and based on their clinical experience, the Scientific Committee prepared two questionnaires that had been proposed in the workshops in April 2024 and January 2025. Each workshop was attended by the same group of participants, while the experts varied depending on the topic discussed. Both participants and Experts were asked to rate all statements with five voting options, as in a Likert scale from 1 to 5: “1 = Strongly Disagree,” “2 = Partially Disagree”, “3 = Neither agree nor disagree”, “4 = Partially Agree”, and “5 = Strongly agree”. The agreement threshold was defined a priori as ≥80% of participants voting “Partially Agree” or “Strongly agree.” To enhance the value of the survey, each question was subsequently discussed in the plenary session to reach a best-practice agreement. The questions are available in Appendix A, Table A1 and Table A2.

### 2.4. Use Cases

Participants were asked to prepare and present 6 paradigmatic use cases from their clinical experience that fit with the following clinical situations:

- a patient with non-visceral progressive disease and CDK 4/6 therapy duration > 24 months, ESR1 not known;
- a patient with visceral progressive disease and CDK 4/6 therapy duration > 24 months;
- a patient with non-visceral progressive disease and CDK 4/6 therapy duration < 12 months;
- a patient with bulky visceral disease after CDK 4/6 therapy, ESR1 mutated;
- a patient with oligometastatic disease in bone, ESR1 mutated;
- a patient with bone and lung disease, CDK 4/6 duration 16 months, ESR1 mutated.

For the purposes of the meeting, use cases were defined as descriptions of how physicians apply an intervention in clinical practice. As such, they did not meet the definition of research involving human subjects and, therefore, did not require ethical approval.

## 3. Results

### 3.1. Survey

The first round of statements addressed the question regarding the factors influencing second-line choice after CDK 4/6 progression; 16 physicians took part in the survey.

**Figure 1:**
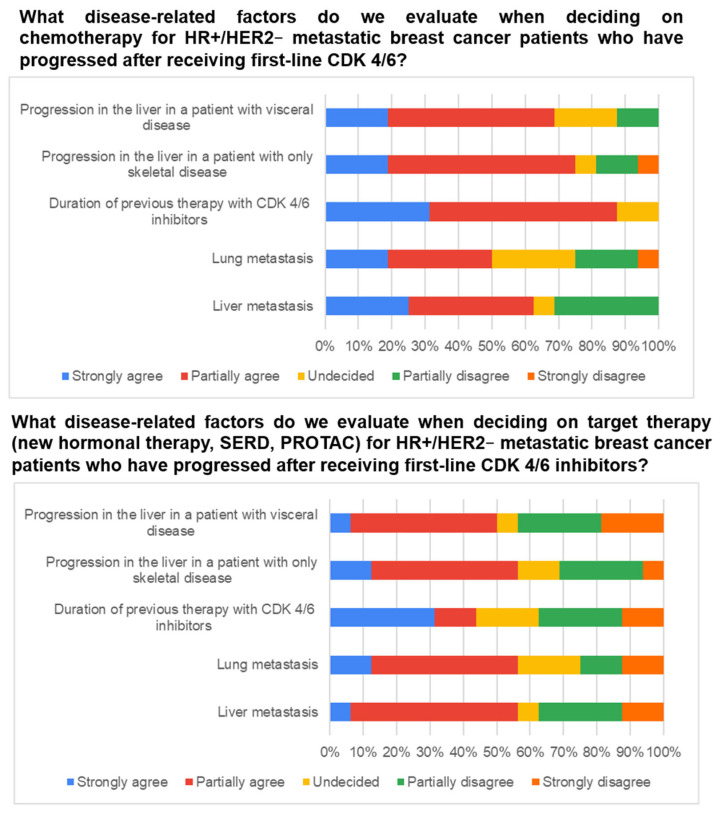
Responses to disease-related questions.

The development of liver metastases at CDK 4/6 progression, either in patients with visceral disease or only with bone metastasis, was a reason to consider CHT after first-line progression: overall, 69% of the participants (11/16) strongly or partially agree on this statement. According to 50% of physicians (8/16), lung metastases at progression addressed the therapeutic choice towards CHT (Figure 1 upper panel). Liver metastases (56%, 9/16) were the main driver of choice for a target therapy, regardless of the presence of previous lesions. The participants showed uncertainty (agreement: 44%, 7/16; disagreement 38%, 6/16) regarding the importance of the duration of first line with CDK4/6 inhibitors (Figure 1 lower panel).

**Figure 2:**
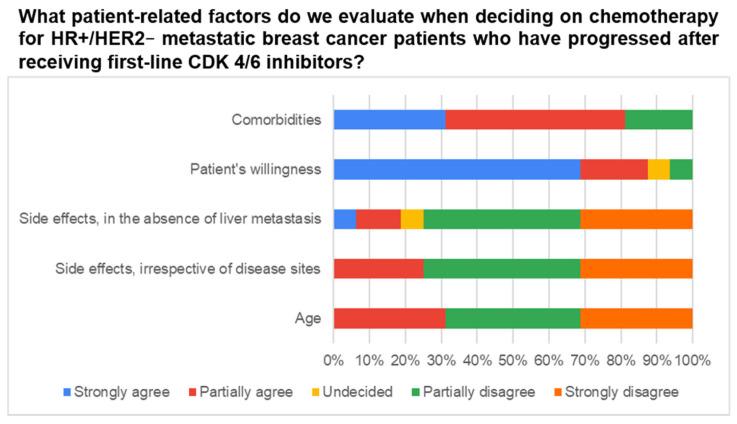
Responses to patient-related questions.

Neither the patient’s age (11/16, 69% strongly or partially disagree), nor side effects occurring during the first-line treatment were considered as conditions for omitting CHT (12/16, 75% strongly or partially disagree). On the contrary, the willingness of patients to receive CHT was reported to be significant (14/16, 88% strongly or partially agree), and even comorbidities may impact the decision between CHT and target therapies (13/16, 81% strongly or partially agree) (Figure 2).

**Figure 3:**
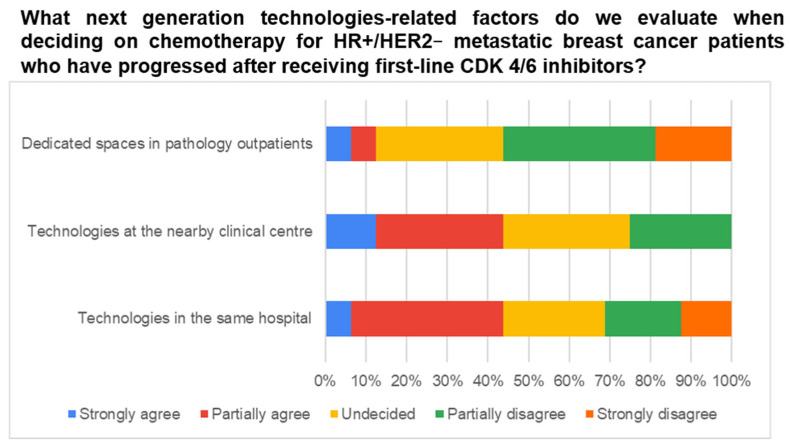
Responses to technology-related questions.

Finally, there was ambiguity in evaluating the influence of the availability of technologies at the hospital or adjacent centers in preferring a targeted therapy. The participants did not consider the technology a limitation for the use of new drugs (Figure 3).

The second round of statements further investigated factors that may affect the choice of the second line of therapy after progression from CDK4/6 inhibitors, including the duration of response and the presence of actionable mutations. Thirty-four participants responded to the questionnaire, held in January 2025, after elacestrant became available in clinical practice.

Almost all participants agreed that both the duration of response and the molecular status were key aspects to consider when choosing a second line of therapy, along with the general clinical condition of the patient (Table 1).

Based on current knowledge and according to guidelines [2], most participants agreed that CHT was not the first option for a second-line treatment of non-visceral disease progression from CDK4/6 inhibitors. However, participants did not agree that genomic testing should be mandatory, irrespective of the duration of previous treatment and relapse sites. For patients with an overt visceral crisis, knowing genomic features would not change the therapeutic choice, and, therefore, it would be redundant to perform the test in all cases. On the contrary, before starting a targeted therapy or enrolling in a clinical study, or in patients with a low performance status, testing should be required. A patient with an endocrine-refractory disease -showing resistance after 3 months- does not realistically achieve a clinical benefit from the therapy, as demonstrated in the CAPItello study, where no clinically significant benefit emerged from treatment with capivasetib + fulvestrant in rapidly progressing patients [19,20].

According to participants’ responses, in patients with oligometastatic progression, monotherapy with fulvestrant may only be appropriate for a small number of very selected patients (less than 5%) and is unlikely to become a second-line standard.

A couple of questions deepened the therapeutic choice according to the duration of response to previous CDK4/6 inhibitor treatment. In case of progression before 12 months from CDK4/6 initiation, 74% of respondents disagreed that CHT was the best therapeutic option: in patients with ESR1 mutation, elacestrant may provide some benefits, if they have not previously received fulvestrant in combination with CDK4/6 inhibitors. A progression within 12 months, however, may suggest secondary resistance, and it is no longer possible to discuss hormone-sensitivity in the second line. In case of progression within 6 months, the discrepancy among participants increased. A six-month exposure to CDK4/6 inhibitors is very limited; in these patients, the mutational status of PIK3CA should be investigated to consider the opportunity to use capivasetib plus fulvestrant, if PIK3CA is mutated, or fulvestrant alone, if PIK3CA is wild type and fulvestrant has not been employed yet.

Almost 80% of participants responded that CHT cannot be considered a valuable option for patients with an ESR1 mutation and a response to CDK4/6 inhibitors shorter than 12 months. ESR1 mutations indicate a disease that is still endocrine-addicted, and an endocrine treatment can be relevant for patients who receive CDK4/6 inhibitors for at least 6 months or have liver metastases. Patients can be treated with elacestrant and followed up closely. During the discussion, however, it emerged that CHT was still an option that should be properly taken into consideration.

Most participants disagreed that the choice of therapy in patients with visceral disease progression was independent of both the duration and response of previous treatment and the presence of mutations. As expected, an agreement was also achieved in considering the mutational status of ESR1 and PIK3CA as essential information for the second-line choice.

### 3.2. Lecture

NF and MU presented an overview of the current scenario of molecular testing of mBC HR+, intending to provide useful information to be translated into clinical practice and promote the discussion among participants on their experience.

Assessing ER and PgR status is pivotal in the management of metastatic breast cancer: tumours positive for both ER and PgR fall into the luminal group and, generally, have a better prognosis than dual-negative receptor tumours [21]. Immunochemistry (ICH) on formalin-fixed, paraffin-embedded (FFPE) tissue sections has been the gold standard method for assessing ER status, but the threshold of positive ER expression is still a matter of debate. In 2021, during the St Gallen International Breast Cancer Conference, the panel did not reach an agreement on cut-off thresholds as it was split fifty-fifty between 1% and 10% [22]. The American Society of Clinical Oncology and the College of American Pathologists (ASCO-CAP) released recommendations for classifying all breast cancers with at least 1% positive cells as HR+ [23]. The cutoff of 1% has a management purpose to define ER positivity and the eligibility for ET. The level of HR expression in BC is variable, as the intensity of expression varies from weak to strong, and the frequency of positive cells varies from 1% to 100%; this has prognostic significance in terms of better outcomes and response to endocrine therapy in BC, in the presence of a strong diffuse nuclear expression [24]. Most HR+ BC show a high IHC score, but approximately 3% of tumours present low ER expression with 1–10% weakly positive cells. These tumours, now referred to as ER-low breast cancer, represent a novel category with a higher histological grade, basal-like expression profiles, and, often, ET resistance [22].

Several critical aspects of the clinical use of ER status were discussed by the panel. ER and PgR are typically assessed concurrently, and the results of HR quantification tests must be reliable, reproducible, and highly predictive. The extent of ER expression in assay results is influenced by tumour cellularity, with lower cellularity often associated with reduced ER levels. While a transition from a luminal to a basal-like phenotype is uncommon, it remains a possibility; therefore, multidisciplinary evaluation is essential to detect such changes.

Based on their clinical experience, participants concurred that biopsy is indispensable both for accurate diagnosis and for selecting the most appropriate therapeutic strategy. Following the initial diagnosis, all patients should undergo biopsy at the first disease recurrence, and approximately 30% should be re-biopsied after the second recurrence. The emergence of new therapeutic options now provides a clear clinical rationale for re-biopsy. Both tissue and liquid biopsies are recognized as valuable tools in clinical practice, each with distinct advantages.

Tissue biopsy offers essential predictive and prognostic information to guide treatment decisions; however, its static nature limits the ability to capture intratumoral heterogeneity and temporal disease evolution under therapeutic pressure. Moreover, repeated tissue sampling can negatively impact patients’ quality of life and is often not feasible [25].

On the other hand, the liquid biopsy can detect driver mutations in circulating tumour DNA (ctDNA) from tumours, is minimally invasive, almost free of complications, and can be repeated over time to monitor disease evolution and guide treatment decisions. Furthermore, it accurately represents tumour heterogeneity, as it may contain DNA from multiple areas of the tumour or metastatic sites [26], but its performance may be negatively affected in case of an insufficient amount of ctDNA with an increased likelihood of obtaining ‘false-negative’. ctDNA concentrations are affected by various factors, including volume, stage, and disease sites: for instance, metastatic disease is associated with increased ctDNA shedding compared to early-stage disease. Some discrepancies may emerge among the results obtained with liquid and tissue biopsy due to tumour heterogeneity, which should be considered seriously when interpreting data [26].

Participants shared the experiences in their hospitals where liquid biopsy was often used to complement tissue biopsy. The discussion highlighted the necessity of setting up a research network to execute tissue and liquid biopsies and aggregate the results to determine the best approach for analyzing each gene.

Liquid biopsy is a valid approach to examine the ESR1 mutational status in patients with metastatic BC. Available evidence in the literature shows a concordance rate for ESR1 mutation between plasma samples and matched tissue ranging from 47 to 100% agreement appears to be lower when plasma samples are compared to archival tissue samples rather than recent tumour biopsies. Despite the limited population size, all series yield consistent findings and endorse the potential of liquid biopsy as a dynamic instrument for detecting genetic alterations in tumours [25].

Almost 30–40% of endocrine-resistant metastatic breast cancer is enriched in ESR1 somatic base pair missense mutations, which can be detected in ctDNA in patients’ blood [27]. ESR1 mutations are rare in primary BC but are becoming more prevalent in metastatic BC; this shift has been linked to the selective pressure of hormone deprivation therapy during the development of endocrine resistance [25]. When compared to patients with wild-type ESR1, these patients with the ESR1 mutation had noticeably inferior clinical outcomes in terms of overall survival and progression-free survival (PFS). For example, somatic ESR1 mutations were not statistically predictive of the outcome of fulvestrant-containing therapy, but they were linked to a shorter PFS following aromatase inhibitor-based treatment. Regarding the various ESR1 variants, the Y537S mutation did not correlate with significantly lower PFS, whereas the D538G mutant did [28].

Given the prognostic significance of ESR1 mutations, the panel emphasized the importance of determining the optimal timing for performing a liquid biopsy to detect mutational changes before radiographic progression becomes evident. As ESR1 mutations emerge during the disease, conducting a liquid biopsy too early may yield a false-negative result due to the absence of the mutation at that stage. Recently, data from the SERENA-6 trial [13] clearly demonstrate that ESR1 mutation can occur early during first-line treatment under the selective pressure of aromatase inhibitor treatment and that switching from AI to Camizestrant, a selective estrogen receptor degrader (SERD) while maintaining CDK 4/6 therapy results in outcome improvement.

Indeed, ESR1 mutations are found in less than 1% of early-stage cases and approximately 5% of advanced-stage cases, with prevalence increasing progressively following first-line and subsequent lines of therapy. Because liquid biopsy is a non-invasive method, the panel recommended its repeated use to enable dynamic molecular profiling and improve the likelihood of detecting ESR1 mutations over time. Drawing from their clinical experience, the panel also noted that the identification of an ESR1 mutation can precede radiologic and clinical progression by at least three months, both in patients with known metastatic disease and during follow-up.

The EMERALD [29,30] and PADA-1 [31] studies used NGS and digital PCR, respectively, to detect the ESR1 mutation, covering the most informative regions of the gene. The European Medicines Agency requires the use of reliable assays, such as NGS or digital PCR, to minimize false-negative results. RT-PCR should be avoided as it is not sensitive enough and cannot detect low-frequency variants. Molecular procedures for liquid biopsy and gene sequencing must be standardized, and the pathologist’s report must include information on the presence of the mutation in a clear and clinically understandable language, avoiding the usage of IUPAC nomenclature, for example.

Lastly, according to the panel, ESR1 is a surrogate of hormone susceptibility, and when CHT is started, there is no need to further search for ESR1 mutations.

Another alteration highly represented in HR+ breast cancer is the upregulation of PI3K/Akt/mammalian target. Activating mutations in PIK3CA occur in ~40% of breast cancers and are drivers for tumorigenesis and tumour progression. Mutated PIK3CA is an actionable target in metastatic settings with alpelisib [32,33,34].

From a diagnostic perspective, adopting a customized methodology to cover all of the clinically significant PI3KCA gene changes in various clinical settings is essential. Because of its cost-effectiveness, sensitivity, specificity, and versatility, RT-PCR is now widely used in almost all molecular pathology laboratories to yield results extremely rapidly. However, RT-PCR lacks standardized procedures and kits to identify all potential PIK3CA mutations, and its multiplexing capability is limited. NGS provides information on the percentage of alleles with the mutation and can simultaneously cover several mutations, even starting with low DNA input. NGS costs are relatively high, but optimizing the laboratory workflow and volumes may generate a favorable cost–benefit ratio [34].

Although tissue is the most suitable sample to identify PI3KCA mutations, liquid biopsy may also be used. In the case that the ctDNA analysis returns a negative result, a second liquid biopsy or, if feasible, a tissue biopsy should be examined. Tissue samples from initial diagnosis are a reliable source for PIK3CA testing, but it is generally preferable (if clinically feasible) to run the test on a biopsy of the metastatic site, as this may best reflect the actual genomic profile of the disease [34].

A survey investigating the real-world routine of PIK3CA testing in HR+ metastatic breast cancer in Italy described a heterogeneous situation in terms of sampling methodology, technical assessment, and clinical reporting for PIK3CA molecular testing [35].

The participants highlighted that PIK3CA mutations do not all have the same predictive value, and attention should be paid to the detection power of the test. In clinical practice, the bottleneck may be the distribution of laboratories nationwide and the limited accessibility to liquid biopsy.

As genes are typically analyzed independently, co-mutations are often overlooked. However, understanding how co-occurring mutations interact and influence treatment response is essential. Discrepancies between liquid and tissue biopsy results can arise, as illustrated by a case shared during the discussion: a patient presented with a PIK3CA mutation detected via tissue biopsy at the metastatic site, while an ESR1 mutation was identified through liquid biopsy. These differences stem from the distinct evaluation parameters of each method—tissue biopsy relies on a laboratory-defined variant allele frequency (VAF) threshold, usually considered clinically relevant above 5%, whereas liquid biopsy requires only the detection of the mutation, regardless of VAF. Such discrepancies, though expected, warrant careful interpretation as they offer valuable insights into the molecular heterogeneity of the disease and its metastatic sites. Moving forward, targeting entire molecular pathways rather than individual gene mutations will be essential. To support this approach, clinical and molecular data must be integrated and jointly evaluated by oncologists and pathologists.

### 3.3. Use Cases

In the rapidly evolving landscape of biomedical research and clinical diagnostics, the inclusion of real-world use cases in educational events serves as a powerful tool to bridge the gap between theory and practice. Use cases provide concrete, context-rich examples that illustrate how complex technologies—such as NGS—are applied in real clinical settings. They not only enhance the reader’s understanding of abstract concepts but also demonstrate the tangible impact of these innovations on patient care, diagnosis, and treatment outcomes. By showcasing specific applications, use cases add credibility, relevance, and depth to scientific discourse, making the narrative more engaging and informative for both expert and non-expert audiences.

Herein, we report some of the use cases included in the discussion.

**Figure 4:**
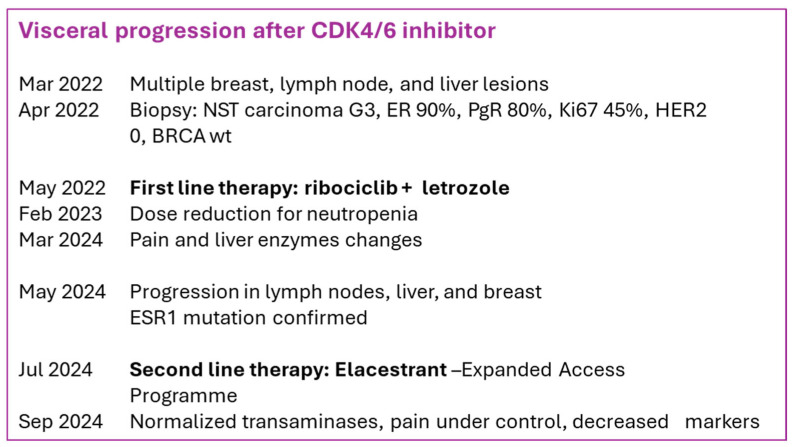
Timeline of the use case describing a patient with visceral progression after a 4-year treatment with ribociclib and fulvestrant, achieving a partial response.

The first use case described a 56-year-old patient, PS 1, with de novo metastatic breast cancer (left breast, multiple lymph nodes and liver), who received first-line therapy with ribociclib and fulvestrant, achieving a partial response with this treatment and subsequently developed visceral progression 4 years later. Mutational analysis revealed an ESR1 mutation, thus making possible the administration of elacestrant (Figure 4).

In a similar patient, the choice between CHT and target therapy is determined by the disease burden and the patient’s biological age. The results from the EMERALD trial [29,30] justified the use of a target therapy, as patients who had progression after 12 months of CDK4/6 treatment achieved a longer PFS; if the organ function is not preserved, target therapy would be chosen with very close monitoring. When the patient has a visceral crisis, and the urgency of intervention is relevant, chemotherapy and, eventually, polychemotherapy are valuable options. In this case, the disease burden, more than the disease site, is a determinant of choice. Chemotherapy can be followed by a targeted therapy like elacestrant [29,30,36] at a later time when organ function is restored. When available, trastuzumab deruxtecan could also be used in case of severe organ dysfunction. Therefore, organ function should be taken into consideration more than the disease site when choosing between chemotherapy and targeted therapy. Chemotherapy may be moved to the next line of treatment based on the available options.

The progression with a bulky visceral disease after CDK4/6 inhibitor treatment was reported in subsequent use cases. In the first example, the disease progressed during adjuvant therapy with aromatase inhibitors at the visceral level, and the patient was symptomatic. Genomic analysis revealed the presence of mutations in ESR1 and PI3K. The second case presented with stage IV disease at onset, harbouring ESR1 mutation; the first line of therapy was ribociclib plus an aromatase inhibitor, which lasted for 24 months, when liver and lymph node metastases occurred.

In the presence of a bulky disease, a rapid clinical response is extremely important, but data on the time-to-response are often missing in clinical trials. Based on panel experience, the clinical outcome of the first line of treatment is reflected in that of the second line, and a disease responsive to CDK inhibitors remains so even if progression occurs in different sites. Usually, the progression after CDK4/6 inhibitors is characterized by high disease burden, but it is unclear what happens at the genomic level. ESR1 mutational status must be ascertained to decide whether and when to use elacestrant in the therapeutic strategy.

**Figure 5:**
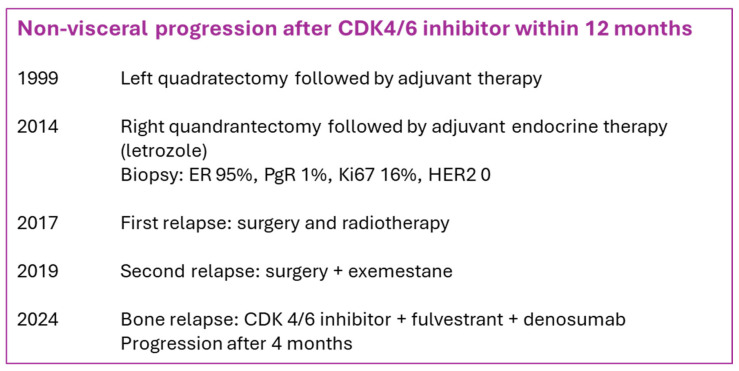
Timeline of the use case describing a patient with non-visceral progressive disease within 12 months of CDK4/6 inhibitor start.

Another use case described a 50-year-old patient, PS 0, diagnosed with ductal carcinoma. After surgery, she received adjuvant chemotherapy. Fifteen years later, the patient required another surgery to remove a ductal carcinoma with cribriform features from the right breast. Radiological evaluation showed no distant metastases, and she was treated with adjuvant letrozole for five years. During this period, the patient developed a cutaneous relapse, which was surgically removed and treated with radiotherapy. In 2019, a second local relapse occurred. She then received adjuvant exemestane until 2024, when bone and lung metastases were detected. The patient (75-year-old) started treatment with a cyclin inhibitor and fulvestrant; however, after four months, progressive skeletal disease was observed. The mutational analysis revealed the presence of an ESR1 mutation (Figure 5).

In patients with progression within 12 months, either non-visceral or visceral, a resistance may occur. Primary and secondary resistances are generally very difficult to define in clinical practice. All mutations identified by NGS or other genomic analysis must be comprehensively considered to choose the best therapeutic option; for instance, the presence of TP53 generates gene and chromosome instability that makes the disease unresponsive to target therapy with a single molecular target [37]. Therefore, treatment should be customized according to the genetic alterations found and, eventually, the patient’s age.

**Figure 6:**
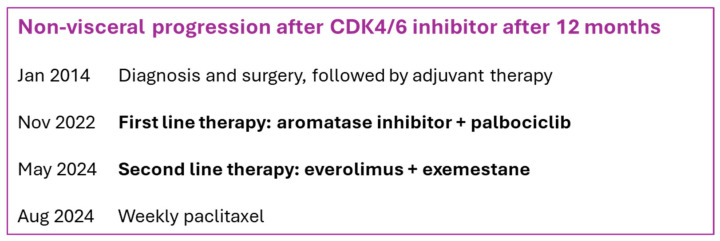
Timeline of the use case describing a patient in progression after the intermediate time of 16 months of CDK4/6 inhibitor treatment.

The last use case illustrated a progression after the intermediate time of 16 months of CDK4/6 inhibitor treatment. The patient was a 46-year-old woman, PS ECOG 0, with no relevant comorbidities and a positive family history of breast cancer (mother diagnosed at the age of 65 years and 1 sister diagnosed at the age of 30 years). She received adjuvant chemotherapy, radiation therapy and endocrine treatment (Tamoxifen + LHRH analogue, switched to Anastrozole after 2 years due to the appearance of endometrial poliposis. After a median Disease Free Interval of 48 months, she developed bilateral breast relapse and lymph node metastases. After the first-line therapy with palbociclib and an aromatase inhibitor, she progressed in the bone and lung. As second-line, the combination of everolimus plus exemestane was chosen which lasted 2.5 months; at progression, the treatment continued with weekly paclitaxel (Figure 6).

From many studies on the resistance to cyclin inhibitors, it emerges that there is a grey area between good and poor responders of patients having inadequate responses and progressing between 12 and 24 months. At present, the concept of time-to-response following treatment with cyclin inhibitors may be overlooked as survival expectations differ depending on endocrine sensitivity. The SERENA-6 trial [38] showed that early detection of ESR1 mutation justifies switching the hormonal treatment and maintaining the CDK4/6 inhibitor in case of a controlled disease. These results could suggest when to test for ESR1 and, eventually, initiate early treatment with elacestrant.

The panel concluded that it is now ineluctable to investigate ESR1 mutational status with liquid biopsy after progression from CDK4/6 inhibitors. The choice between liquid and tissue biopsy depends on several factors: liquid biopsy is more sensitive to analyze ESR1 mutations or a polyclonal disease and is preferable when metastases are in hard-to-reach or bone sites [39]. Nonetheless, it must learn to re-biopsy as early and as much as possible. A single biopsy is not representative, as, to date, no biomarkers can unravel the heterogeneity of the disease.

During the discussion, the participants highlighted the occurrence of a further case in clinical practice: the patient who presents a double mutation in PIK3CA and ESR1. These cases should be deeply examined and collected in specific and large case series. In the EMERALD study, about 39% (62/159) of patients showed ESR1-PI3KCA co-mutation with a worse prognosis in terms of progression-free survival compared to other populations treated with elacestrant. Double mutations may be present in the same clone or two different clones; ESR1 mutations may have developed during CDK4/6 inhibition [40]. Hypothesizing a therapeutic strategy, patients previously treated with fulvestrant could be tested for ESR1 and treated with elacestrant [41]. Real-life data or very large studies are warranted to validate such a strategy and clarify the better sequencing.

## 4. Conclusions

The Hermione Exchange Educational Program provided the opportunity to debate the current state of HR+ metastatic breast cancer management. The scenario is rapidly evolving thanks to both the advances of technologies to investigate the disease’s mutational status and the availability of new therapeutic options, such as elacestrant, and several challenges will arise in the future.

However, this project has some limitations. The findings are based on expert opinion derived from a survey approach conducted within a limited number of workshops, which may introduce selection bias and restrict the generalizability of the results. The number of participants involved in the voting process varied across meetings, potentially affecting the consistency of the consensus. In addition, the use cases were descriptive in nature and not based on systematically collected data, thus limiting their strength as evidence. Finally, the rapidly evolving therapeutic landscape of HR+ metastatic breast cancer may impact the long-term applicability of these findings.

Tissue and liquid biopsy are powerful tools to describe tumour molecular features over time; such complexity should be harnessed by a close dialogue between oncologists, molecular biologists, and pathologists to optimize the therapeutic choice according to the mutational status of patients. Therefore, genomic testing is recommended at diagnosis and should be repeated during treatment to monitor the disease. As it is now possible to exploit the feasibility of liquid biopsy, all possible efforts should be made to make genomic testing nationwide and timely accessible.

The response to the previous line with CDK4/6 inhibitors and the duration of treatment are further determinants of choice for the second line. In this perspective, the clinical experience acquired over the years must be integrated with new molecular knowledge but remains essential for the proper management of the patient. Further real-world data and other discussions like that occurred during the Hermione Program are warranted to establish the valuable sequencing after the first line with CDK4/6 inhibitors according to patients’ and disease characteristics.

## Figures and Tables

**Table 1:** Questionnaire on factors addressing the choice towards CHT or target therapy in patients with HR+ metastatic breast cancer who have progressed after the first line of treatment with CDK 4/6 inhibitors (January 2025). Possible answers were: strongly agree, partially agree, undecided, partially disagree, strongly disagree.

In patients with non-visceral disease progression after CDK 4/6 inhibitors, the choice of second-line treatment is closely linked to the duration of previous treatment.	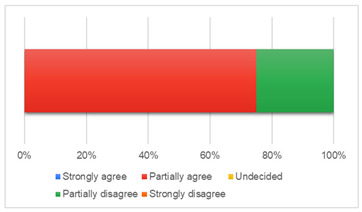
In patients with non-visceral disease progression after CDK 4/6 inhibitors, the choice of second-line treatment is closely linked to molecular biology results	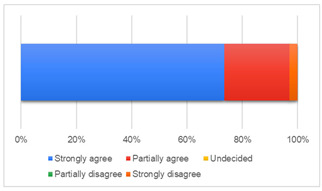
In patients with non-visceral progression of disease after CDK 4/6 inhibitors, the choice of second-line treatment is CHT	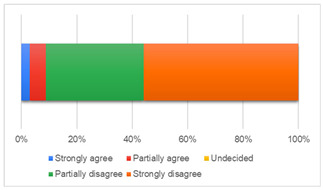
In patients with disease progression after CDK 4/6 inhibitors, genomic testing is mandatory, irrespective of the duration of previous treatment and relapse sites.	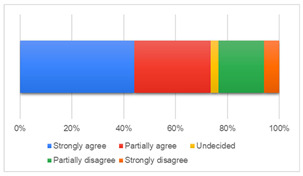
In patients with oligometastatic progression after treatment with CDK 4/6 inhibitors, the choice in clinical practice falls on fulvestrant.	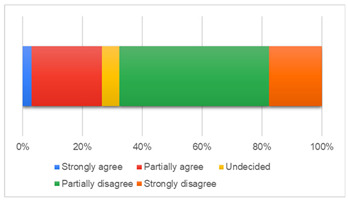
In patients with disease progression after CDK 4/6 inhibitors, the duration of previous treatment of less than 12 months requires the use of CHT.	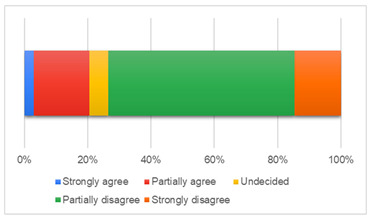
In patients with disease progression after CDK 4/6 inhibitors, the duration of previous treatment of less than 6 months assumes the use of CHT.	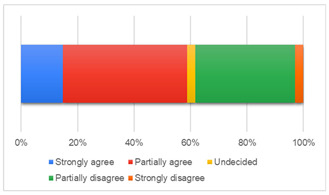
In patients with disease progression after CDK 4/6 inhibitors, the duration of previous treatment of less than 12 months presupposes the use of CHT, even in the presence of mutated ESR1.	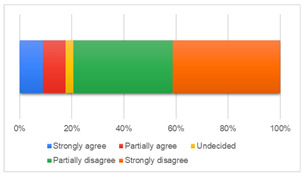
In patients with visceral disease progression after CDK 4/6 inhibitors, the choice of second-line treatment is closely related to the duration of previous treatment. The choice of second-line treatment depends on the duration of response.	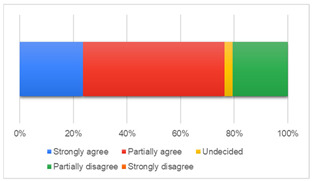
In patients with visceral progression of disease after CDK 4/6 inhibitors, the choice of 2-line treatment is closely related to the duration of previous treatment, regardless of the presence of actionable mutations.	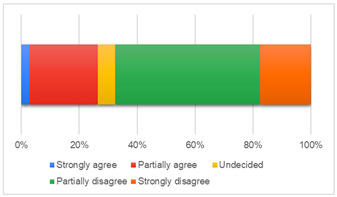
For a therapeutic decision in 2-line patients after CDK 4/6 inhibitor failure, the mutational status of ESR1 and PIK3CA must always be known.	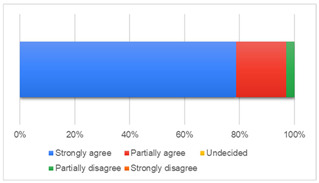

## Data Availability

Data sharing is not applicable to this article as no datasets were generated or analyzed during the current study.

## Abbreviations

The following abbreviations are used in this manuscript:

CHT	Chemotherapy
CDK	Cyclin-dependent kinases
ER	Estrogen receptor
ET	Endocrine therapy
HR	Hormone receptor
PgR	Progesterone receptor

## Appendix A

**Table A1:** Questionnaire on factors addressing the choice towards CHT or target therapy in patients with HR+ metastatic breast cancer who have progressed after the first line of treatment with CDK 4/6 inhibitors (April 2024). Possible answers were: strongly agree, partially agree, undecided, partially disagree, strongly disagree.

**1. What disease-related factors do we evaluate when deciding on CHT for HR+/HER2- metastatic breast cancer patients who have progressed after receiving first-line CDK 4/6 inhibitors?**
Presence of visceral disease (liver metastasis)
Presence of visceral disease (lung metastasis)
Duration of previous therapy with CDK 4/6 inhibitors shorter than median of clinical studies (approximately 24 months)
Progression in the liver in a patient with only skeletal disease
Progression in the liver in a patient with visceral disease
**2. What disease-related factors do we evaluate when deciding on target therapy (new hormonal therapy, SERD, PROTAC) for HR+/HER2- metastatic breast cancer patients who have progressed after receiving first-line CDK 4/6 inhibitors?**
Presence of visceral disease (liver metastasis)
Presence of visceral disease (lung metastasis)
Duration of previous therapy with CDK 4/6 inhibitors shorter than median of clinical studies (approximately 24 months)
Progression in the liver in a patient with only skeletal disease
Progression in the liver in a patient with visceral disease
**3. What patient-related factors do we evaluate when deciding on CHT for HR+/HER2- metastatic breast cancer patients who have progressed after receiving first-line CDK 4/6 inhibitors?**
The patient’s age is a condition for omitting CHT, regardless of disease sites
Side effects occurring during previous treatment with CDK 4/6 inhibitors, in particular neutropenia, are a condition for omitting CHT, irrespective of disease sites
Side effects occurring during previous treatment with CDK 4/6 inhibitors, in particular neutropenia, are a condition for omitting CHT, only in the absence of liver metastasis
The patient’s wish is a condition for omitting CHT
The patient’s comorbidities are a condition for omitting CHT
**4. What next generation technologies (NGS, WGS, etc** **.** **)-related factors do we evaluate when deciding on CHT for HR+/HER2- metastatic breast cancer patients who have progressed after receiving first-line CDK 4/6 inhibitors?**
The availability of the technologies in the same hospital is a condition for addressing the choice towards a target therapy
The availability of technologies at nearby clinical centres is a condition for directing the choice towards a target therapy
The availability of dedicated space in pathology outpatient clinics is a condition for guiding the choice towards a target therapy

**Table A2:** Questionnaire on factors addressing the choice towards second line of therapy in patients with HR+ metastatic breast cancer in progression after CDK 4/6 inhibitors (January 2025). Possible answers were: strongly agree, partially agree, undecided, partially disagree, strongly disagree.

In patients with non-visceral disease progression after CDK 4/6 inhibitors, the choice of second-line treatment is closely linked to the duration of previous treatment.
In patients with non-visceral disease progression after CDK 4/6 inhibitors, the choice of second-line treatment is closely linked to molecular biology results
In patients with non-visceral progression of disease after CDK 4/6 inhibitors, the choice of second-line treatment is CHT
In patients with disease progression after CDK 4/6 inhibitors, genomic testing is mandatory, irrespective of duration of previous treatment and relapse sites
In patients with oligometastatic progression after treatment with CDK 4/6 inhibitors, the choice in clinical practice falls on fulvestrant
In patients with disease progression after CDK 4/6 inhibitors, duration of previous treatment of less than 12 months requires the use of CHT
In patients with disease progression after CDK 4/6 inhibitors, the duration of previous treatment of less than 6 months assumes the use of CHT
In patients with disease progression after CDK 4/6 inhibitors, the duration of previous treatment of less than 12 months presupposes the use of CHT, even in the presence of mutated ESR1
In patients with visceral disease progression after CDK 4/6 inhibitors, the choice of second-line treatment is closely related to the duration of previous treatment. The choice of second-line treatment depends on the duration of response
In patients with visceral progression of disease after CDK 4/6 inhibitors, the choice of 2-line treatment is closely related to the duration of previous treatment, regardless of the presence of actionable mutations
For a therapeutic decision in 2-line patients after CDK 4/6 inhibitor failure, the mutational status of ESR1 and PIK3CA must always be known

## References

[1] AIRTUM I numeri del Cancro 2025. https://www.aiom.it/wp-content/uploads/2025/12/2025_NDC_web.pdf.

[2] Gennari A., André F., Barrios C.H., Cortés J., de Azambuja E., DeMichele A., Dent R., Fenlon D., Gligorov J., Hurvitz S.A. (2021). ESMO Clinical Practice Guideline for the diagnosis, staging and treatment of patients with metastatic breast cancer. Ann. Oncol..

[3] de Azambuja E., Barrios C.H., Bartsch R., Curigliano G., Dent R., Gennari A., Loi S., Paluch-Shimon S., Pistilli B., Saura C. (2026). Metastatic breast cancer: ESMO Clinical Practice Guideline for diagnosis, treatment and follow-up. Ann. Oncol..

[4] Gradishar W.J., Moran M.S., Abraham J., Aft R., Agnese D., Allison K.H., Anderson B., Burstein H.J., Chew H., Dang C. (2022). Breast Cancer, Version 3.2022, NCCN Clinical Practice Guidelines in Oncology. J. Natl. Compr. Canc Netw..

[5] Hilton H.N., Clarke C.L., Graham J.D. (2018). Estrogen and progesterone signalling in the normal breast and its implications for cancer development. Mol. Cell Endocrinol..

[6] Höller A., Nguyen-Sträuli B.D., Frauchiger-Heuer H., Ring A. (2023). Diagnostic and Prognostic Biomarkers of Luminal Breast Cancer: Where are We Now?. Breast Cancer.

[7] Zhang L., Sun F., Wang X., Zhao W., Jia Y., Wang H., Han Y., Tong Z. (2026). Efficacy and safety of treatments in HR+/HER2- advanced breast cancer after CDK4/6 inhibitor progression: A network meta-analysis and scoping review. BMC Cancer.

[8] Rugo H.S., Curigliano G., Cescon D.W., Penault-Llorca F., Harbeck N., Im S.A., Park Y.H., Barrios C., Modi S., Tolaney S.M. (2026). New perspectives on endocrine therapy suitability for hormone receptor-positive metastatic breast cancer in clinical practice. Breast.

[9] Verret B., Bottosso M., Hervais S., Pistilli B. (2022). The Molecular Predictive and Prognostic Biomarkers in Metastatic Breast Cancer: The Contribution of Molecular Profiling. Cancers.

[10] André F., Ciruelos E., Rubovszky G., Campone M., Loibl S., Rugo H.S., Iwata H., Conte P., Mayer I.A. (2019). Alpelisib for PIK3CA-Mutated, Hormone Receptor–Positive Advanced Breast Cancer. N. Engl. J. Med..

[11] Buonaiuto R., Fordellone M., Caltavituro A., Cataldo M.L., Criscitiello C., Dieci M.V., Lambertini M., Botticelli A., Giuliano M., Giordano A. (2025). Efficacy and safety of systemic therapies following progression on CDK4/6 inhibitors in HR+/HER2- metastatic breast cancer: A systematic review and network meta-analysis. eClinicalMedicine.

[12] Guglielmi G., Del Re M., Gol L.S., Bengala C., Danesi R., Fogli S. (2024). Pharmacological insights on novel oral selective estrogen receptor degraders in breast cancer. Eur. J. Pharmacol..

[13] Bidard F.C., Mayer E.L., Park Y.H., Janni W., Ma C., Cristofanilli M., Bianchini G., Kalinsky K., Iwata H., Chia S. (2025). First-Line Camizestrant for Emerging ESR1-Mutated Advanced Breast Cancer. N. Engl. J. Med..

[14] Gombos A., Goncalves A., Curigliano G., Bartsch R., Kyte J.A., Ignatiadis M., Awada A. (2023). How I treat endocrine-dependent metastatic breast cancer. ESMO Open.

[15] Bardia A., Hu X., Dent R., Yonemori K., Barrios C.H., O’Shaughnessy J.A., Wildiers H., Pierga J.Y., Zhang Q., Saura C. (2024). Trastuzumab Deruxtecan after Endocrine Therapy in Metastatic Breast Cancer. N. Engl. J. Med..

[16] Fontes-Sousa M., Amorim M., Salta S., Palma De Sousa S., Henrique R., Jerónimo C. (2019). Predicting resistance to endocrine therapy in breast cancer: It’s time for epigenetic biomarkers. Oncol. Rep..

[17] Wright T.M., Wardell S.E., Jasper J.S., Stice J.P., Safi R., Nelson E.R., McDonnell D.P. (2014). Delineation of a FOXA1/ERα/AGR2 regulatory loop that is dysregulated in endocrine therapy-resistant breast cancer. Mol. Cancer Res..

[18] Raheem F., Karikalan S.A., Batalini F., El Masry A., Mina L. (2023). Metastatic ER+ Breast Cancer: Mechanisms of Resistance and Future Therapeutic Approaches. Int. J. Mol. Sci..

[19] Turner N.C., Oliveira M., Howell S.J., Dalenc F., Cortes J., Gomez Moreno H.L., Hu X., Jhaveri K., Krivorotko P., Loibl S. (2023). Capivasertib in Hormone Receptor-Positive Advanced Breast Cancer. N. Engl. J. Med..

[20] Dilawari A., Buturla J., Osgood C., Gao X., Chen W., Ricks T.K., Schaefer T., Avasarala S., Reyes Turcu F., Pathak A. (2024). FDA Approval Summary: Capivasertib With Fulvestrant for HR-Positive, HER2-Negative Advanced Breast Cancer With PIK3CA/AKT1/PTEN Alterations. J. Clin. Oncol..

[21] Pescia C., Guerini-Rocco E., Viale G., Fusco N. (2023). Advances in Early Breast Cancer Risk Profiling: From Histopathology to Molecular Technologies. Cancers.

[22] Fusco N., Ragazzi M., Sajjadi E., Venetis K., Piciotti R., Morganti S., Santandrea G., Fanelli G.N., Despini L., Invernizzi M. (2021). Assessment of estrogen receptor low positive status in breast cancer: Implications for pathologists and oncologists. Histol. Histopathol..

[23] Allison K.H., Hammond M.E.H., Dowsett M., McKernin S.E., Carey L.A., Fitzgibbons P.L., Hayes D.F., Lakhani S.R., Chavez-MacGregor M., Perlmutter J. (2020). Estrogen and Progesterone Receptor Testing in Breast Cancer: ASCO/CAP Guideline Update. J. Clin. Oncol..

[24] Rakha E.A., Tse G.M., Quinn C.M. (2023). An update on the pathological classification of breast cancer. Histopathology.

[25] Urso L., Vernaci G., Carlet J., Lo Mele M., Fassan M., Zulato E., Faggioni G., Menichetti A., Di Liso E., Griguolo G. (2021). ESR1 Gene Mutation in HR-Positive HER2-Negative Metastatic Breast Cancer: Concordance Between Tumor Tissue and ctDNA. Front. Oncol..

[26] Russo A., Incorvaia L., Del Re M., Malapelle U., Capoluongo E., Gristina V., Castiglia M., Danesi R., Fassan M., Giuffrè G. (2021). Molecular profiling of solid tumors by liquid biopsy: Position paper of Italian Scientific Societies. ESMO Open.

[27] Li Z., Wu Y., Yates M.E., Tasdemir N., Bahreini A., Chen J., Levine K.M., Priedigkeit N.M., Nasrazadani A., Ali S. (2022). Hotspot ESR1 Mutations Are Multimodal and Contextual Modulators of Breast Cancer Metastasis. Cancer Res..

[28] Zhang K., Hong R., Xu F., Xia W., Kaping L., Qin G., Zheng Q., Lu Q., Shi Y.X., Yuan Z.Y. (2018). Clinical value of circulating ESR1 mutations for patients with metastatic breast cancer: A meta-analysis. Cancer Manag. Res..

[29] Bidard F.C., Kaklamani V.G., Neven P., Streich G., Montero A.J., Forget F., Mouret-Reynier M.A., Sohn J.H., Taylor D., Harnden K.K. (2022). Elacestrant Versus Standard Endocrine Therapy for ER-Positive, HER2-Negative Advanced Breast Cancer: EMERALD Trial. J. Clin. Oncol..

[30] Bardia A., Cortés J., Bidard F.C., Neven P., Garcia-Sáenz J., Aftimos P., O’Shaughnessy J., Lu J., Tonini G., Scartoni S. (2024). Elacestrant in ER+, HER2- Metastatic Breast Cancer with ESR1-Mutated Tumors: Subgroup Analyses from EMERALD. Clin. Cancer Res..

[31] Callens C., Bidard F.C., Curto-Taribo A., Trabelsi-Grati O., Melaabi S., Delaloge S., Hardy-Bessard A.C., Bachelot T., Clatot F., De La Motte Rouge T. (2022). Real-Time Detection of ESR1 Mutation in Blood by ddPCR in PADA-1: Feasibility and Cross-Validation with NGS. Anal. Chem..

[32] Venetis K., Pepe F., Munzone E., Sajjadi E., Russo G., Pisapia P., Ivanova M., Bonizzi G., Vacirca D., Rappa A. (2022). Analytical Performance of NGS and RT-PCR on FFPE Tumor Tissues for PIK3CA Testing in HR+/HER2- Breast Cancer. Cells.

[33] André F., Ciruelos E.M., Juric D., Loibl S., Campone M., Mayer I.A., Rubovszky G., Yamashita T., Kaufman B., Lu Y.S. (2021). Alpelisib plus fulvestrant for PIK3CA-mutated HR+/HER2- advanced breast cancer: Final OS results from SOLAR-1. Ann. Oncol..

[34] Fusco N., Malapelle U., Fassan M., Marchiò C., Buglioni S., Zupo S., Criscitiello C., Vigneri P., Dei Tos A.P., Maiorano E. (2021). PIK3CA Mutations as a Molecular Target for HR+/HER2- Metastatic Breast Cancer. Front. Oncol..

[35] Pepe F., Venetis K., Cursano G., Frascarelli C., Pisapia P., Vacirca D., Scimone C., Rappa A., Russo G., Mane E. (2024). PIK3CA testing in HR+/HER2- metastatic breast cancer: Real-world data from Italian laboratories. Pharmacogenomics.

[36] Rugo H.S., Kaklamani V., McArthur H., Wander S.A., Gradishar W., Mahtani R., Pegram M., Lustberg M., Swallow E., Maitland J. (2026). Real-World Outcomes of Elacestrant in ER+, HER2-, ESR1-Mutant Metastatic Breast Cancer. Clin. Cancer Res..

[37] Aftimos P., Oliveira M., Irrthum A., Fumagalli D., Sotiriou C., Gal-Yam E.N., Robson M.E., Ndozeng J., Di Leo A., Ciruelos E.M. (2021). Genomic and Transcriptomic Analyses of Breast Cancer Primaries and Matched Metastases in AURORA. Cancer Discov..

[38] Turner N., Huang-Bartlett C., Kalinsky K., Cristofanilli M., Bianchini G., Chia S., Iwata H., Janni W., Ma C.X., Mayer E.L. (2023). Design of SERENA-6, a phase III switching trial of camizestrant in ESR1-mutant breast cancer. Future Oncol..

[39] Bello O., Rieur O., Vidula N. (2026). The use of cfDNA/ctDNA to guide treatment selection in metastatic breast cancer. Pharmacogenomics.

[40] O’Leary B., Cutts R.J., Liu Y., Hrebien S., Huang X., Fenwick K., André F., Loibl S., Loi S., Garcia-Murillas I. (2018). Genetic Landscape and Clonal Evolution of Resistance to Palbociclib plus Fulvestrant in PALOMA-3. Cancer Discov..

[41] Lloyd M.R., Weipert C.M., Ali A., Solomon S.R., Saha J., Lipsyc-Sharf M.D., Hamilton E.P., Kalinsky K., Brufsky A.M., Bardia A. (2026). Clinical and Genomic Factors Associated with Elacestrant Outcomes in ESR1-Mutant Metastatic Breast Cancer. Clin. Cancer Res..

